# A Novel Potent Crystalline Chitin Decomposer: Chitin Deacetylase from *Acinetobacter schindleri* MCDA01

**DOI:** 10.3390/molecules27165345

**Published:** 2022-08-22

**Authors:** Guang Yang, Yuhan Wang, Yaowei Fang, Jia An, Xiaoyue Hou, Jing Lu, Rongjun Zhu, Shu Liu

**Affiliations:** 1 Jiangsu Key Laboratory of Marine Bioresources and Environment, Jiangsu Ocean University, Lianyungang 222005, China; 2 Co-Innovation Center of Jiangsu Marine Bio-Industry Technology, Jiangsu Ocean University, Lianyungang 222005, China; 3 Jiangsu Marine Resources Development Research Institute, Jiangsu Ocean University, Lianyungang 222000, China; 4 College of Food Science and Engineering, Jiangsu Ocean University, Lianyungang 222005, China

**Keywords:** chitin deacetylase, crystalline chitin, *Acinetobacter schindleri*, uraD_N-term-dom superfamily, chitosan

## Abstract

Chitosan is a functional ingredient that is widely used in food chemistry as an emulsifier, flocculant, antioxidant, or preservative. Chitin deacetylases (CDAs) can catalyze the hydrolysis of acetyl groups, making them useful in the clean production of chitosan. However, the high inactivity of crystalline chitin catalyzed by CDAs has been regarded as the technical bottleneck of crystalline chitin deacetylation. Here, we mined the *AsCDA* gene from the genome of *Acinetobacter schindleri* MCDA01 and identified a member of the uraD_N-term-dom superfamily, which was a novel chitin deacetylase with the highest deacetylation activity. The *AsCDA* gene was expressed in *Escherichia coli* BL21 by IPTG induction, whose activity to colloidal chitin, α-chitin, and β-chitin reached 478.96 U/mg, 397.07 U/mg, and 133.27 U/mg, respectively. In 12 h, the enzymatic hydrolysis of *As*CDA removed 63.05% of the acetyl groups from α-chitin to prepare industrial chitosan with a degree of deacetylation higher than 85%. *As*CDA, as a potent chitin decomposer in the production of chitosan, plays a positive role in the upgrading of the chitosan industry and the value-added utilization of chitin biological resources.

## 1. Introduction

Chitin, a linear homopolymer of β-1,4-glycosidically linked N-acetylglucosamine (GlcNAc) residues, is the second largest natural polymer renewable resource after cellulose. Some 6 to 8 million tons of chitin solid waste are generated during the fishery processing of crab, shrimp, and lobster, which are value-added processing by-products of aquatic foods [1,2,3]. However, natural chitin has a dense crystalline structure, a highly ordered three-dimensional network, and a high degree of polymerization and insolubility in regular solvents, which severely limits the development and commercial application of chitin [4,5,6]. Therefore, natural chitin bioresource is often considered to be a burdensome waste product of the marine and biotechnology industries [7]. Chitosan, the deacetylated derivative of chitin, prepared from natural chitin sources with a degree of deacetylation higher than 55%, is widely used in the food industry, medicine, environmental protection, and agriculture due to its safety, non-toxic, biodegradable, biocompatibility, anti-oxidation, and bacteriostatic properties [8,9,10]. Significantly, chitosan is most widely used in the food industry, and these characteristics and the chemical structure of chitosan may contribute to its functionality and application as an emulsifier, flocculant, antioxidant, or preservative in food chemistry. Meanwhile, chitosan preparations have been accepted as food additives and functional food ingredients in China, Japan, and Korea. 

Chitin deacetylases (CDAs, EC 3.5.1.41) catalyze the deacetylation of chitin to prepare chitosan and chitin nanofibers to realize the value-added utilization of chitin biological resources [2,11,12]. Compared to the traditional physical and chemical methods to prepare chitosan from chitin, the bio-enzymatic production process treated by microbial CDAs has the advantages of safety, controllability, an environmentally friendly process, and high efficiency [13]. Significantly, the production of chitosan by this enzymatic process of CDAs resulted in a higher molecular weight, homogeneous end products, and the desired degree of acetylation, which is more useful in food chemistry and has a higher economic value [14]. Chitin deacetylases, classified in the carbohydrate esterase family 4 (CE4), were mostly reported in fungi and some insect species, and seldom reported in bacteria. Since the CDA from *Mucor rouxii* (*Mr*CDA) was first discovered in 1975, CDAs have been studied in terms of fermentation optimization, physical and chemical properties, development and application, structure, and function [15,16,17]. 

Previous studies have reported only eight CDAs from microbial sources that catalyze the deacetylation of crystalline chitin (see Supplementary Table S1) [7,11,18,19,20]. These CDAs with low activity catalyzing crystalline chitin were the bottlenecks that limited the industrial production of chitosan by CDA enzymatic hydrolysis. CDAs with high activity on crystalline chitin can meet the demand for the industrial production of chitosan by enzymatic treatment. To obtain better accessibility to the acetyl groups for the deacetylation, natural chitin was pretreated and modified through various physical and chemical methods such as sonicating, grinding, heating, derivatization, and interaction with saccharides [21,22]. A variety of methods have been studied on improving the efficiency of enzymatic hydrolysis to crystalline chitin [19,23,24,25,26,27]. CDAs are, however, currently commercially unavailable. Therefore, the identification of a bacterial CDA with high activity on crystalline chitin should be explored to enzymatically convert it into chitosan.

*Acinetobacter schindleri* MCDA01 was screened producing the highest enzyme activity of wild CDA enzyme with 201.37 U/mL, which had a high efficiency of enzymatic hydrolysis to crystalline chitin for preparation of chitin nanofibers with significantly increased length and width by catalytic crystal chitin [11]. Although the wild CDA from *A. schindleri* MCDA01 has recently been enzymologically characterized, the genome sequence of *A. schindleri* MCDA01 and the gene sequence of its CDA are still unclear. Meanwhile, the activity of CDA from *A. schindleri* MCDA01 toward crystalline chitin has not yet been evaluated. In the present study, the genomic DNAs of *A. schindleri* MCDA01 were sequenced and analyzed, and the *CDA* gene (*AsCDA*) was mined based on the genomic analysis. The characterization and application of *As*CDA toward crystalline chitin were evaluated. The preparation system of chitosan by the enzymatic method of *As*CDA was constructed, which will play a positive role in the chitosan industrial technology upgrade and quality improvement. This study will perfect the enzymatic basis for the clean product of chitosan by CDAs and provide an experimental basis for the molecular mechanism of the deacetylation of crystalline chitin catalyzed by *As*CDA.

## 2. Results and Discussion

### 2.1. General Genomic Features of the A. schindleri MCDA01 Strain

**Figure 1:**
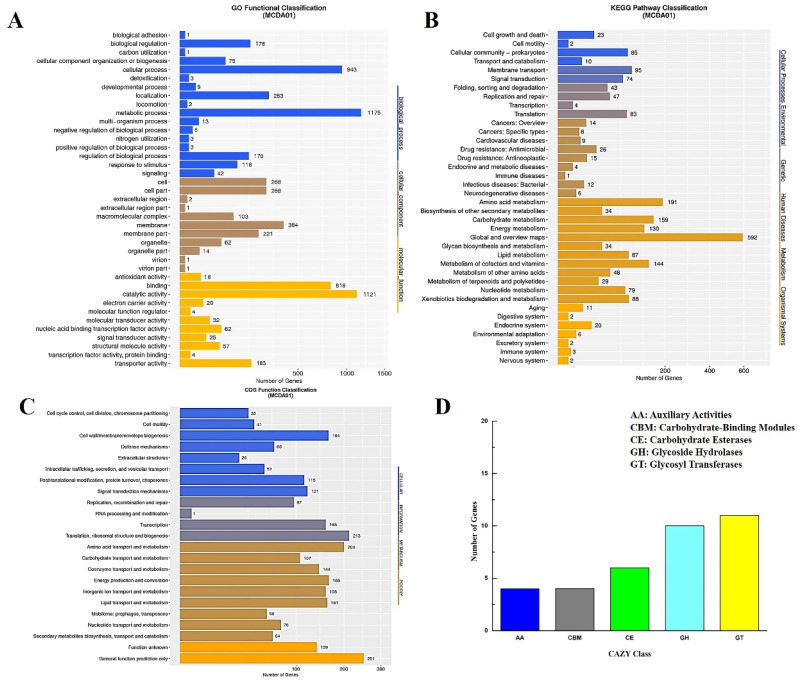
Genome analysis of *Acinetobacter schindleri* MCDA01: (**A**) Histogram showing the distribution of Gene Ontology (GO) terms; (**B**) Eucaryotic Orthologous Groups (COG) functional gene classification; (**C**) The Kyoto Encyclopedia of Genes and Genomes (KEGG) function annotation; (**D**) Carbohydrate-Active Enzymes (CAZy) family distribution map.

*Acinetobacter schindleri* MCDA01 has been reported to produce CDAs with higher activity for the preparation of chitin nanofibers with a notable increase in length and width by catalytic crystal chitin [11]. The purification process of the wild enzyme of CDA from *A. schindleri* MCDA01 is complex and the enzyme activity is seriously lost. This study focused on the clean production of chitosan through recombinant CDA from *A. schindleri* MCDA01 catalyzed chitin deacetylation, which will enhance the chitosan industrial technology upgrade and quality improvement. Consequently, the genome of *A. schindleri* MCDA01 was sequenced and analyzed to further research and acquire in-depth genomic level insights related to its molecular evolutionary, functional metabolite, and biosynthetic potential. Sequencing the whole genome of the *A. schindleri* MCDA01 strain showed that the total length of the genome was 3.33 Mb (including the plasmid genome 0.17 Mb) with 43% GC content (see Supplementary Figure S1). The whole genome of *A. schindleri* MCDA01 encoded 3107 putative predicted genes, which were 84.31% of the genome sequence including 21 ribosomal rRNAs, 88 tRNAs, and 9 sRNAs (Table 1). The function annotation of putative CDs was successfully performed on 1902 GO genes (61.21% of the genome sequence, Figure 1A), 1881 KEGG genes (60.57% of the genome sequence, Figure 1B), 2267 COG genes (72.96% of the genome sequence, Figure 1C), and 35 CAZy genes (1.12% of the genome sequence, Figure 1D). To further analyze whether the *A. schindleri* MCDA01 strain was a potential chitin decomposer, chitin degrading enzymes involved in chitin deacetylation and hydrolysis were identified by the CAZy database. The 11 genes were annotated as deacetylases or chitinases including five gene-encoding chitinases and six gene-encoding deacetylases (including *As*CDA) (see Supplementary Table S2), which were annotated for carbohydrate metabolic processes and catalytic and hydrolase activity toward carbon–nitrogen. The genome size, GC content, and CD quantity of *A. schindleri* MCDA01 were larger than other reported strains of *A. schindleri*, which may mean that more genes of the *A. schindleri* MCDA01 genome involved in the microbial metabolism process could adapt to different environmental conditions (see Supplementary Table S3) [28].

### 2.2. Comparative Genome Analysis of Acinetobacter schindleri MCDA01

**Figure 2:**
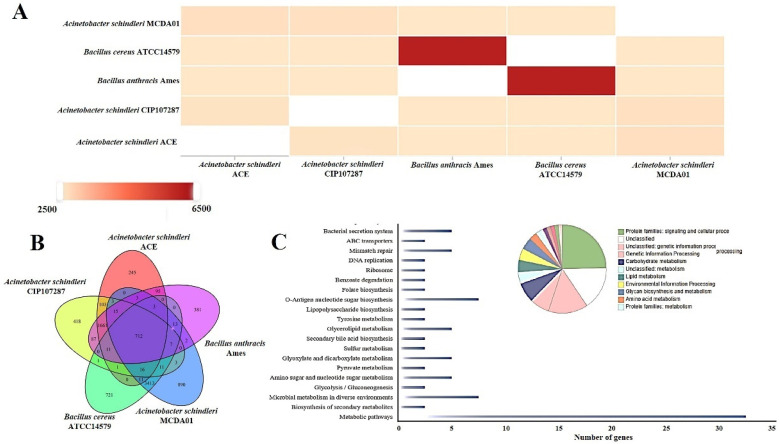
Comparative genome analysis of *Acinetobacter schindleri* MCDA01, *Acinetobacter schindleri* CIP107287, *Acinetobacter schindleri* ACE, *Bacillus anthracis* Ames, and *Bacillus cereus* ATCC14579: (**A**) similarity relations of genomes between four strains; (**B**) unique genes of the genomes of four strains; (**C**) KEGG pathway classification of these unique genes from the genome of *Acinetobacter schindleri* MCDA01.

To investigate the evolutionary specificity and diversity of *A. schindleri* MCDA01, we carried out a comparative genomic analysis of different strains. Comparative genomics analysis revealed that *A. Schindleri* MCDA01 has few homologous genes with *B. anthracis* Ames and *B. cereus* ATCC14579, but more homologous genes with *A. schindleri* CIP107287 and *A. schindleri* ACE (Figure 2A) and these results were largely consistent with the previous study of Ye et al. [11]. Particularly, 418 genes (13.45% of the genome sequence) were shared, and unique genes of *A. schindleri* MCDA01 strain (Figure 2B) were involved in signaling and cellular processing, genetic information processing, carbohydrate metabolism (including *As*CDA), lipid metabolism, environmental information processing, glycan biosynthesis and metabolism, amino acid metabolism, and so on (Figure 2C), which displayed that *A. Schindleri* MCDA01 has higher carbohydrate metabolism activity and is a potent chitin decomposer [29,30].

### 2.3. Genome Mining and Bioinformatics Analysis of AsCDA

**Figure 3:**
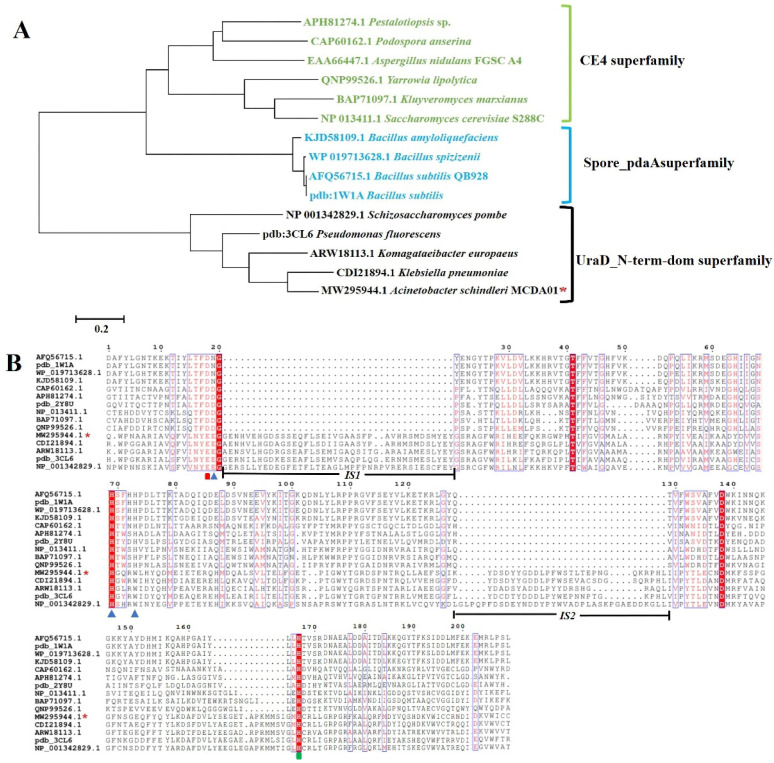
Bioinformatics analysis of *As*CDA: (**A**) phylogenetic analysis of neighbor-joining phylogenetic tree; (**B**) multiple amino acid sequence alignment: the metal binding triads are indicated with blue triangles; the catalytic base and catalytic acid are indicated with red rectangles and green rectangles, respectively.

There are four genes annotated deacetylases belonging to the CE4 family in the *A. schindleri* MCDA01 genome database, and *As*CDA was mined uniquely according to the molecular mass 31 kDa of natural chitin deacetylase [11]. The sequences of *As*CDA were uploaded to GenBank with Accession NO. MW295944.1, but not opened. The *AsCDA* gene is a 975 bp encoding 324 amino acids with a Polysacc_deac_1 domain. To further determine the phylogenetic status of *As*CDA, we performed a multiple sequence alignment, phylogenetic analysis, and homologous modeling of the *As*CDA amino acid sequence with other 14 members of the deacetylase superfamily. The phylogenetic analysis identified these deacetylases from three different clades representing the CE4 superfamily, spore_pdaA superfamily, and uraD_N-term-dom superfamily, respectively [31,32]. *As*CDA is clustered together with other members of the uraD_N-term-dom superfamily and has high sequence similarity with *Schizosaccharomyces pombe Sp*CDA, polysaccharide deacetylase from *Pseudomonas aeruginosa*, CDA from *Rhodococcus equi F6,* and PuuE protein from *Pseudomonas fluorescens*, some of which had enzymatic hydrolysis to chitooligosaccharides or chitin pretreated by [TBA][OH] (Figure 3A) [31,32]. Interestingly, multiple sequence alignment of these enzymes revealed that *As*CDA has a few notable differences from other CDAs of the CE4 superfamily. The conserved Asp-His-His metal-binding triad of the CE4 family is replaced by Glu-His-Trp of *As*CDA (active sites: Glu^51^, Glu^52^, His^140^, Trp^144^, and His^276^; Glu^51^: base catalytic site; His^276^: acid catalytic site, and the catalytic amino acid residues are different, with Glu replacing Asp for the acidic amino acid) (Figure 3B). Two conserved inserted segments (IS1 and IS2) of the *As*CDA sequence do not have a counterpart in the CE4 superfamily (Figure 3B). In the next section, we will analyze the crystal structure of *As*CDA to explore and elucidate the enzymatic reaction mechanism of this arrangement in the *As*CDA sequence. Importantly, these results indicate that *As*CDA is a new PuuE homolog of the uraD_N-term-dom superfamily and has a high-enzyme activity to catalyze crystalline chitin deacetylation.

### 2.4. Heterologous Expression of AsCDA in Escherichia coli

**Figure 4:**
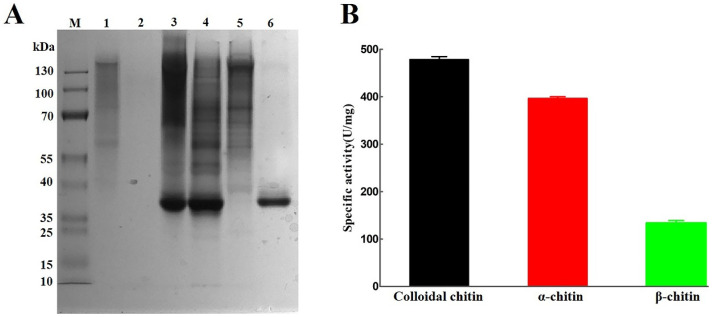
Purification and characterization of the recombinant protein *As*CDA from *Acinetobacter schindleri* MCDA01: (**A**) heterologous expression and purification of *As*CDA were analyzed by SDS-PAGE on a 12% gel after Coomassie brilliant blue R-250 staining; (**B**) specific activity of purified *As*CDA to colloidal chitin, α-chitin, and β-chitin. Lane M, proteins marker with standard molecular Masses; lane 1, negative control (empty vector pET-28a); lane 2, Fermentation supernatant from *Escherichia coli* BL21 containing expression vector pET28a-AsCDA induced with IPTG; lane 3, insoluble phase of cellular extracts from *Escherichia coli* BL21 containing expression vector pET28a-AsCDA induced with IPTG; lane 4, soluble phase of cellular extracts from *Escherichia coli* BL21 containing expression vector pET28a-AsCDA induced with IPTG; lane 5, effluent fractions when all proteins were washed clean with elution 80 mM imidazole conc; lane 6, purified *As*CDA with elution 300 mM imidazole.

The recombinant plasmid pET-28a-*AsCDA* was a heterologous expression in *E. coli* BL21. SDS-PAGE analysis showed that the molecular weight of purified *As*CDA was approximately 37.5 kDa with 2.11 mg/mL by nickel affinity chromatography (Figure 4A), which was expected on the MW of natural chitin deacetylase produced by *A. schindleri* MCDA01. Some previous studies observed that the MW of chitin deacetylases ranged from 15 to 100 kDa and most chitin deacetylases were in the range of 35-40 kDa [8,14,22,33,34]. The specific activity of purified *As*CDA treated colloidal chitin, α-chitin and β-chitin were 478.96 U/mg, 397.07 U/mg, and 133.27 U/mg by acetic acid assay kit, respectively (Figure 4B). Previous results have shown that most CDAs had a high activity on chitooligosaccharides, water-soluble chitosan, and colloidal chitin, but low or no activity toward crystalline chitin [2,30]. Thus, *As*CDA has potential application in the effective deacetylation of crystalline chitin substrates, especially α-chitin.

### 2.5. Deacetylation of Crystalline Chitin Catalyzed by Chitin Deacetylase AsCDA

**Figure 5:**
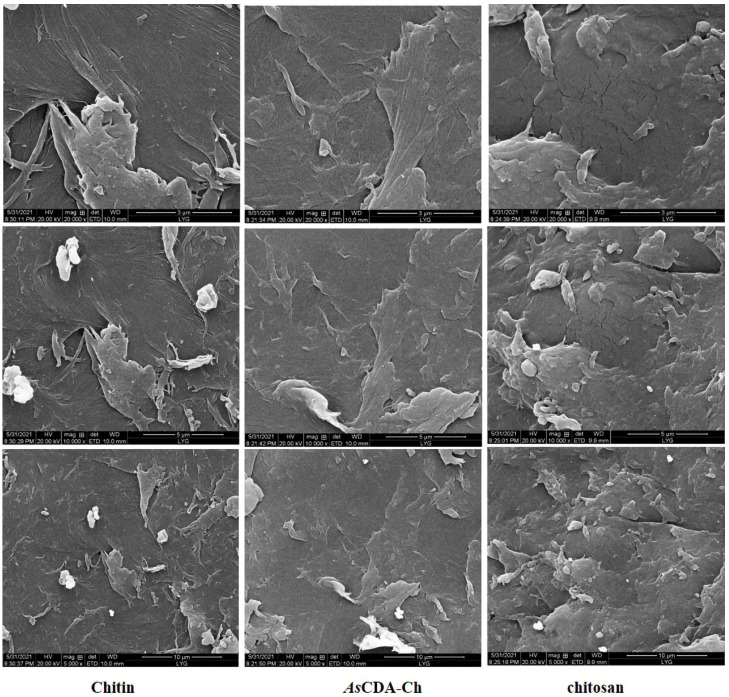
SEM images of untreated chitin, chitin treated by *As*CDA, and chitosan with magnificent ×20,000 times, ×10,000 times, and ×5000 times.

To determine characterized microstructure changes, chitosan, α-chitin, and *As*CDA-Ch were performed for Fourier transform infrared spectroscopy (FT-IR) and scanning electron microscope (SEM). The results of SEM, as in Figure 5, show that untreated α-chitin was large dense particles stacked in a layered structure whose surface compactly arrange a crystalline microfibril structure, which was compact, rough, and texture. The bulk structure of *As*CDA-Ch, however, was severely damaged. Holes and dense rifts were observed on the surface of *As*CDA-Ch and its fibers became blurred and indistinct and exhibited a less well-defined interface with separated sheets. The particle state of *As*CDA-Ch was similar to chitosan, and was obviously different from α-chitin. The microstructure images of α-chitin and chitosan by SEM were reported in previous studies, which further verified that deacetylation of α-chitin by *As*CDA was similar to chitosan [35,36,37]. The reason for microstructure changes in α-chitin treated by *As*CDA was the reduction in acetyl group content, which caused the intramolecular and molecular structure to be destroyed or weakened [38].

**Figure 6:**
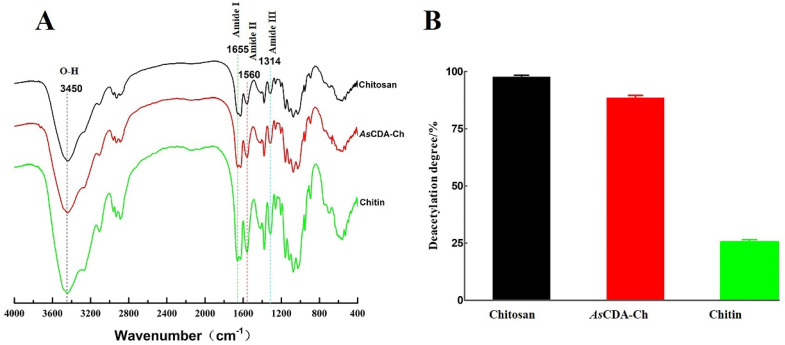
FT-IR spectra and determination of the degree of deacetylation of chitosan (black), *As*CDA treated chitin (red) and chitin (green): (**A**) FT-IR spectra; (**B**) determination of the degree of deacetylation.

**Figure 7:**
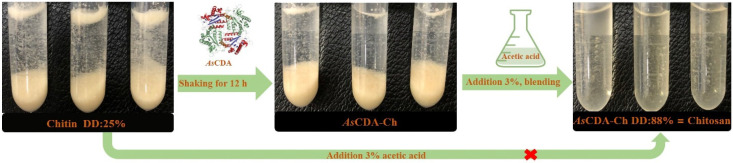
Simplified diagram of *As*CDA to catalyze crystalline chitin deacetylation to prepare chitosan.

The comparative analysis results of chitosan, α-chitin, and *As*CDA-Ch by FT-IR showed that the structure of the three samples was basically the same, according to the similarity of the peak position and absorbance. The amide I, II, and III bands of the characteristic peaks were observed at 1655, 1560, and 1314 cm^−1^ in the FT-IR spectrum, and the peak position of 1655 cm^−1^ indicated that the samples were deacetylated products of α-type chitin (Figure 6A) [39]. From the absorption bands at 1655 cm^−1^ (amide I) and 3450cm^−1^ (-OH group) emerged the displacement of the absorption peak in different chitin samples. The A_1655_/A_3450_ ratio linear with the degree of deacetylation of chitin was used to calculate the degree of deacetylation (DD) of samples [40]. The DD of the samples was determined on the basis of the A_1655_/A_3450_ value from FT-IR results, the degree of deacetylation of α-chitin, *As*CDA-Ch, and chitosan were 25.44%, 88.49%, and 97.54%, respectively (Figure 6B). *As*CDA removed 63.05% of the acetyl groups for α-chitin pretreated for 12 h, and the degree of deacetylation did not change with the increase in treatment time (Figure S3). The results indicated that the enzymatic hydrolysis of *As*CDA resulted in the release of an acetyl group and increased the degree of deacetylation of α-chitin. Compared to the *Na*CDA from *Nitratireductor aquimarinus* MCDA3-3 studied by our group, the *As*CDA showed a significant improvement in reaction time and substrate concentration of the catalytic preparation of chitosan [35]. Chitosan was the deacetylated derivative of chitin with a high rate of deacetylation higher than 55% [2]. *As*CDA has the highest activity toward deacetylation of crystalline chitin and it removes the acetyl group content of crystalline chitin, which had not been pretreated through various physical and chemical methods until this current study (see Supplementary Figure S1) (Figure 7). The enzymatic method is a promising alternative to these traditional physical and chemical methods to promote the chitosan industrial technology upgrade and quality improvement [7]. *As*CDA is a potent chitin decomposer to catalyze chitin deacetylation to prepare chitosan and realize the value-added utilization of chitin biological resources. 

## 3. Materials and Methods

### 3.1. Materials

*Acinetobacter schindleri* MCDA01 (CGMCC NO.13539)-producing chitin deacetylase was isolated from marine mud collected from Yanwei Harbor (34°29′0″ N, 119°47′0″ E), Lianyungang City, China, which was laboratory preservation strains [11]. Operationally, the medium for the cell growth of *A. schindleri* MCDA01 was a 2216E broth at 20 °C and 180 rpm for 48 h. The α-chitin was kindly provided by Professor Fan Yimin from Nanjing Forestry University, which was purified from swimming crab (*Portunus trituberculatus*) shells collected from Nantong, a seaside city in Jiangsu Province, China. Chitosan was the degree of deacetylation greater than 95% and average molecular weight (MW) of approximately 2 × 10^5^, which was purchased from Sangon Biotech (Shanghai, China). All other reagents and solvents were analytical grade.

### 3.2. Isolation, Sequencing, and De Novo Assembly of the Acinetobacter schindleri MCDA01 Genome

The genomic DNA was extracted by Bacteria Genomic DNA Extraction Kit (TaKaRa, Dalian, China) according to the manufacturer’s recommended protocol. Sequencing libraries were generated using a Next Ultra DNA Library Prep Kit for Illumina (New England Biolabs Ltd). The library quality was assessed on the Qubit@ 2.0 Fluorometer (Thermo Scientific) and Agilent Bioanalyzer 2100 system. Subsequently, the libraries with high-quality reads were sequenced using a HiSeq 2500 sequence platform provided by the Illumina company and PE125 strategies. A preliminary assembly of the filtered valid data was performed using SOAP*denovo2* (v.2.04.4; SOAPdenovo2, RRID: SCR_014986) [41,42,43]. Whole-genome data of *A. schindleri* MCDA01 was finally submitted to GenBank under accession number CP076603, Bioproject PRJNA733816, and Biosample SAMN19459915.

### 3.3. Gene Prediction and Annotation

After obtaining the whole-genome data of *A. schindleri* MCDA01, genes were predicted using a GeneMarkS v.4.28 (http://topaz.gatech.edu/GeneMark/ (accessed on 14 July 2022)) [44]. The function of putative coding sequences (CDs) was annotated via local BLAST searches against NCBI NR and SwissProt databases. The genome blast search (E value less than 1E-5, minimum alignment length ≥ 40%, matching similarity ≥ 40%) was deposited in diverse protein databases, including the Kyoto Encyclopedia of Genes and Genomes (KEGG) (http://www.kegg.jp/kegg/tool/annotate_sequence.html (accessed on 14 July 2022)) for the metabolic pathways, Non-Redundant Protein Database (NR, ftp://ftp.ncbi.nih.gov/blast/db/FASTA/nr.gz (accessed on 14 July 2022)) for protein alignments, the SwissProt (ftp://ftp.ebi.ac.uk/pub/databases/uniprot/knowledgebase/uniprot_sprot.fasta.gz (accessed on 14 July 2022)) for mapping the gene-ontology terms, Eucaryotic Orthologous Groups (KOG) for eukaryotic clusters of orthologues, and Gene Ontology (GO, http://www.geneontology.org/ (accessed on 14 July 2022)) for annotation of the homologous genes and their function, location of cellular components, and biological processes [25,45,46,47,48,49].

### 3.4. Comparative Genome Analysis and Gene Mining of Chitin Deacetylase

To determine the diversity and strain-specific features in *A. schindleri* MCDA01, the pan-genome was analyzed and searched for orthologous genes in the genome of different bacterial strains (*Acinetobacter schindleri* MCDA01, *Acinetobacter schindleri* CIP107287, *Acinetobacter schindleri* ACE, *Bacillus anthracis* Ames, and *Bacillus cereus* ATCC14579, genome information see Supplementary Table S2) with the software package GET_HOMOLOGUES v3.2.1, which can cluster homologous gene families using the bidirectional best-hit, COG triangles, or OrthoMCL v2.0 clustering algorithms [50,51,52]. For *Acinetobacter schindleri* CIP107287 and *A. schindleri* ACE, the most closely related to strain *A. schindleri* MCDA01, there were no known reports on producing chitin deacetylase [53,54]. *Bacillus anthracis* Ames and *B. cereus* ATCC14579, producing chitin deacetylase, were complete draft genomes [55,56]. The whole genome synteny between the genome pairs was performed by using the MUMmer-4.0.0beta2 with the ‘-mum’ parameter [57]. In our previous studies, the molecular mass of natural chitin deacetylase was approximately 31 kDa produced by *A. schindleri* MCDA01, which was an important basis for the gene mining of *As*CDA [11]. The putative CDAs were identified using a dbCAN2 meta-server for annotation of carbohydrate-active enzymes (CAZymes) [45]. A DNAMAN v9.0.1.116 (https://www.lynnon.com/index.html) was used for amino acid sequence alignment and conservative domain analysis. Phylogenetic analysis indicating the relationship of *As*CDA to other chitosanases was performed using MEGA 6 software (see Supplementary File).

### 3.5. Heterologous Expression and Purification of AsCDA in Escherichia coli BL21

The cDNA of the *AsCDA* gene was amplified by PCR using the *As*CDA1-F (*Bam*HI:GGATCCACAACTGCTTACTTACGCGGA) and *As*CDA1-R (*Hin*dIII:AAGCCTGTCCTGGTGCGCAGGAAAATG), verified by DNA sequencing, cloned into the pET-28a expression vector and transformed into *Escherichia coli* BL21 (TransGen Biotech, Beijing, China). The expression of the recombinant *As*CDA was induced by the addition of isopropyl b-D-thiogalactoside (IPTG, final concentration of 1mM) in Luria–Bertani medium (LB medium, containing 50 ng/mL kanamycin) [58]. After overnight induction at 16 °C, the bacterial cells were collected, resuspended in lysis solution, and sonicated for 30 min. The sediment and supernatant were collected after centrifugation for 20 min (4 °C, 12,000 rpm/min). Recombinant *As*CDA was purified by the AKTA-purifier protein purification system and Histidine Ni-Sepharose™ Fast Flow, analyzed by SDS-PAGE on a 12% gel after Coomassie brilliant blue R-250 staining. Protein concentration was determined by the bicinchoninic acid method using BSA as a standard. 

### 3.6. Enzyme Assay of AsCDA

The enzymatic activity assay of *As*CDA was performed as Acetic Acid Assay Kit (Acetate kinase analyzer format, Megazyme, Republic of Ireland) described by Bergmeyer with modification [59]. The Standard curve of acetic acid with ∆A_340_ was drawn for enzyme assay of *As*CDA (Figure S2). The reaction mixture of 2 mg α-chitin and 10 µL *As*CDA (2.11 µg/µL) was added to 190 µL phosphate buffer (pH 7.0) and incubated at 30 °C for 30 min; the enzyme reaction was terminated by boiling at 100 °C for 10 min in a sealed tube prior. The supernatant was collected by centrifugation at 12,000 rpm for 5 min, and the amount of acetic acid in 10 µL supernatant was determined to calculate the enzyme activity through the A_340_ according to the assay procedure of the Acetic Acid Assay Kit. The minimum differential absorbance of the Acetic Acid Assay Kit was 0.005 absorbance unit, which was equivalent to 0.063 mg/L acetic acid in the sample solution. One unit of *As*CDA activity was defined as the amount of enzyme required to catalyze the release of 1µmol acetic acid per minute. 

### 3.7. Scanning Electron Microscope Observation of Deacetylation Effect

Chitosan, α-chitin, and α-chitin treated by *As*CDA (*As*CDA-Ch) were characterized microstructure changes by Scanning electron microscopy (Alexandridis, Ghasemi, Furlani, & Tsianou). *As*CDA-Ch was a final concentration of 30 g/L chitin pretreated by *As*CDA enzyme solution with 50 mM phosphate buffer with shaking fermentation at 30 °C for 12 h (treatment times were set at 0 h, 2 h, 4 h, 6 h, 8 h, 10 h, 12 h, 14 h, 16 h, 18h, 22 h, and 24 h. After 12 h, the degree of deacetylation did not change with the increase in treatment time). All samples were dried to a constant weight at 100 °C and were respectively adhered to the metal sample stage with a conductive adhesive, and a metal film was sprayed in a vacuum evaporator and then observed under a scanning electron microscope at 20 kV [19,37].

### 3.8. Deacetylation Was Measured by Infrared Spectroscopy

Chitosan, α-chitin, and *As*CDA-Ch carried out an infrared spectrometer in a wavenumber region of 400 to 4000 cm^−1^ using FT-IR spectra (Nicolet-iS10), which was based on previously described work by Chai et al. [35]. In the FT-IR spectrum of the enzymatic chitin, the absorption bands at 1659^−1^, 1560^−1^, and 1314 cm^−1^, respectively, were the amide I, II, and III bands of the characteristic peaks of acetamido groups, and the absorbance bands at 1655 cm^−1^ and 3450 cm^−1^ used to determinate of the degree of deacetylation [60,61]. The difference in DD before (α-chitin) and after (*As*CDA-Ch) enzyme treatment of *As*CDA toward α-chitin was the removed acetyl groups. Infrared determination of the degree of deacetylation calculation formula:

(1) $$\mathrm{DD}=\left(1-\frac{A_{1655}/A_{3450}\;}{1.33}\right)\times 100$$

### 3.9. Statistical Analyses

All tests were performed in triplicate and data were presented as the mean ± SE. One-way ANOVA and Duncan’s multiple range tests were used to test for variance and significant differences using the SPSS software (version 26.0).

## 4. Conclusions

Chitosan is a biodegradable, biocompatible, and nontoxic aminopolysaccharide, which is widely used in food chemistry as an emulsifier, flocculant, and functional food additive. Chitin deacetylases catalyzed the crystal chitin deacetylation to prepare chitosan, which was a safe, controllable, and environmentally friendly process to replace the traditional current chemical methods. The low catalytic activity of CDAs for the deacetylation of crystalline chitin, however, did not meet the demand for the industrial production of chitosan. Thus, appropriate CDA should be explored to improve the efficiency of enzymatic hydrolysis to crystalline chitin for chitosan production. In this study, *As*CDA, a member of the uraD_N-term-dom superfamily, was mined uniquely through the *A. schindleri* MCDA01 genome database of bioinformatics analysis. *As*CDA was biochemically characterized in *E. coli* BL21, which has a high activity rate and deacetylation reaction for crystalline chitin. The results of FT-IR and SEM confirmed that the enzymatic hydrolysis of *As*CDA released the acetyl group and increased the degree of deacetylation of crystalline chitin, which removed 63.05% of the acetyl groups for crystalline chitin and prepared industrial chitosan in 12 h. *As*CDA is a potent chitin decomposer to catalyze chitin deacetylation to prepare chitosan, and these findings will hopefully realize the effective conversion of chitin bioresources into their valuable derivatives: chitin nanofibers, chitosan, and chitooligosaccharides. The study on crystal structures of *As*CDA will be a suitable method to elucidate the molecular mechanism of the deacetylation of long-chain chitin catalyzed by CDAs, which, in the future, will be the next scientific research subject of our research group.

## Figures and Tables

**Table 1:** General characteristics of the *Acinetobacter schindleri* MCDA01 genome.

General Features	*A. schindleri* MCDA01
Genome size (bp)	3,332,387
GC content (%)	43
Gene number	3107
Gene length	2,809,674
% of Genome (genes)	84.31
Gene average length	904.3
rRNAs	21
tRNAs	88
sRNAs	9
CAZy assignment	35
COG assignment	2267
GO assignment	1902
KEGG alignment	1881

## Supplementary Materials

The following supporting information can be downloaded at: https://www.mdpi.com/article/10.3390/molecules27165345/s1, Figure S1: Circular graph of the *Acinetobacter schindleri* MCDA01 complete genome; Figure S2: Standard curve of acetic acid for enzyme assay of *As*CDA; Figure S3: The determination of the degree of deacetylation of α-chitin treated by *As*CDA with different time points; Table S1: CDAs with characterized activity on crystalline chitin; Table S2: Chitin degrading enzymes in *Acinetobacter schindleri* MCDA01 identified by the CAZy database; Table S3: General genome features of seventeen strains in this study.
