# Supplementary material for: A Novel Potent Crystalline Chitin Decomposer: Chitin Deacetylase from Acinetobacter schindleri MCDA01
Source: Molecules. 2022 Aug 22;27(16):5345. doi: 10.3390/molecules27165345 (PMC9416191; doi:10.3390/molecules27165345)
Supplement: Supplementary file 1 [file molecules-27-05345-s001.zip › molecules-1842087-supplementary.pdf]

A novel potent crystalline chitin decomposer: chitin deacetylase from *Acinetobacter schindleri* MCDA01

Guang Yang [a,b,c,d], Yuhan Wang[a,d], Yaowei Fang [a,b,c,d], Jia An[a,d], Xiaoyue Hou [a,b,c,d], Jing Lu [a,b,c,d], Rongjun Zhu[a,d], Shu Liu [a,b,c,d *]

[a] *Jiangsu Key Laboratory of Marine Bioresources and Environment, Jiangsu Ocean University, Lianyungang, 222005, China*

[b] *Co-Innovation Center of Jiangsu Marine Bio-industry Technology, Jiangsu Ocean University, Lianyungang 222005, China*

[c] *Jiangsu Marine Resources Development Research Institute, Jiangsu Ocean University, Lianyungang, 222000, China*

[d] *College of food science and engineering, Jiangsu Ocean University, Lianyungang, 222005, China*

⁎ Corresponding author:

Shu Liu: E-mail: jdliushu@163.com

Tel.: +860515861246008

Fax: +860515861246008

Supplementary Table S1：

Table S1 CDAs with characterized activity on crystalline chitin.

Enzyme	Organism	GenBank No.	PDB ID	Deacetylation of crystalline chitin	Reference
*As*CDA	*Acinetobacter schindleri*	MW295944.1	-	63.05%	This study
*An*CDA	*Aspergillus nidulans*	EAA66447.1	2Y8U	0.50%	23
*Sc*CDA2	*Saccharomy cescerevisiae*	NM001182196.1	-	9.16%	20
*Ar*CE4	*Arthrobacter* sp.	LT630322.1	5LFC	0.01%	55
*Cl*CDA	*Colletotrichum lindemuthianum*	AAT68493.1	2IW0	0.50%	19
*Sb*CDA	*Scopulariopsis brevicaulis*	-	-	3.7%	55
NaCDA	*Nitratireductor aquimarinusMCD*	-	-	32.37%	48
MeCDA	*Microbacterium esteraromaticum*	-	-	32.75%	18

Supplementary Table S2：

Table S2 Chitin degrading enzymes in *Acinetobacter schindleri* MCDA01 identified by the CAZy database.

Function	Gene ID	Enzymes	CAZy family	Number of genes
Deacetylase	MCDA01GL002828	N-acetylglucosamine deacetylase	CE11	1
	MCDA01GL001582	Diacetylchitobiose deacetylase	CE14	1
	MCDA01GL002213	Acetylxylan esterase	CE3	1
	MCDA01GL002854	Chitin deacetylase	CE4	3
	MCDA01GL002176			
	MCDA01GL002179			
Hydrolase	MCDA01GL001269	Chitinases of classes I, II, and IV	GH19	1
	MCDA01GL000141	Modules of approx for chitinases	CBM50	4
	MCDA01GL000167			
	MCDA01GL001985			
	MCDA01GL002353			

Supplementary Table S3:

Table S3 General genome features of seventeen strains in this study.

Strain	Level	Size (Mb)	GC %	Scaffolds	CDs	BioSample	BioProject
Acinetobacter schindleri HZE30-1	Complete	3.33719	42.5	9	3023	SAMN12478519	PRJNA558439
Acinetobacter schindleri HZE23-1	Complete	3.37611	42.7	11	3053	SAMN12478518	PRJNA558439
Acinetobacter schindleri HZE33-1	Complete	3.3207	42.6	9	3004	SAMN12478520	PRJNA558439
Acinetobacter schindleri SGAir0122	Complete	3.24466	42.6	2	2826	SAMN08222738	PRJNA388547
Acinetobacter schindleri ACE	Complete	3.26805	42.6	7	2959	SAMN04956557	PRJNA320745
Acinetobacter schindleri H3	Complete	3.2587	42.5	5	2904	SAMN09464373	PRJNA477264
Acinetobacter schindleri CIP 107287	Scaffold	3.39456	42.3	18	3081	SAMN01828163	PRJNA183293
Acinetobacter schindleri S00243	Scaffold	3.17198	42.7	65	2895	SAMN14908301	PRJNA632052
Acinetobacter schindleri GTC3703	Contig	3.24409	42.4	98	2965	SAMD00019892	PRJDB1754
Acinetobacter schindleri DRN	Contig	3.15797	42.7	105	2848	SAMN08942761	PRJNA450622
Acinetobacter schindleri NIPH900	Scaffold	3.4149	42.4	12	3096	SAMN01087915	PRJNA183303
Acinetobacter schindleri TG19614	Contig	3.19703	42.4	159	2914	SAMN02471245	PRJNA172718
Acinetobacter schindleri UBA3129	Scaffold	2.76116	42.7	309	0	SAMN06456231	PRJNA348753
Acinetobacter schindleri UBA10059	Scaffold	2.51164	41.2	290	2284	SAMN08020004	PRJNA417962
Acinetobacter schindleri MCDA01	Complete	3.33239	43.1	1	3107	SAMN19459915	PRJNA733816
Bacillus anthracis Ames	Complete	5.22729	35.4	2	5072	SAMN02603432	PRJNA309
Bacillus cereus ATCC10876	Scaffold	5.99608	34.8	2	5753	SAMN02736987	PRJNA243525

Supplementary Figure S1：

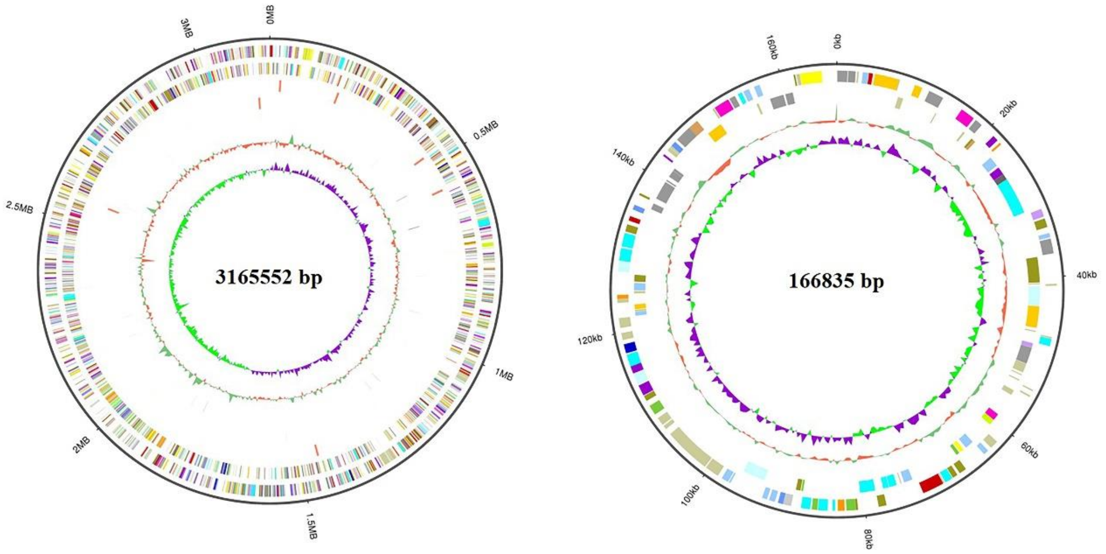

Figure S1 Circular graph of the *Acinetobacter schindleri* MCDA01 complete genome. Circles (from inside to outside): circle 1, GC skew; circle 2, GC content; circle 3, tRNA and rRNA genes on the reverse strand; circles 4, tRNA and rRNA genes on the forward strand; circle5, protein-coding genes on the forward reverse strand; circle 6, protein-coding genes on the forward strand; circle 7, genome location information.

Supplementary Figure S2：

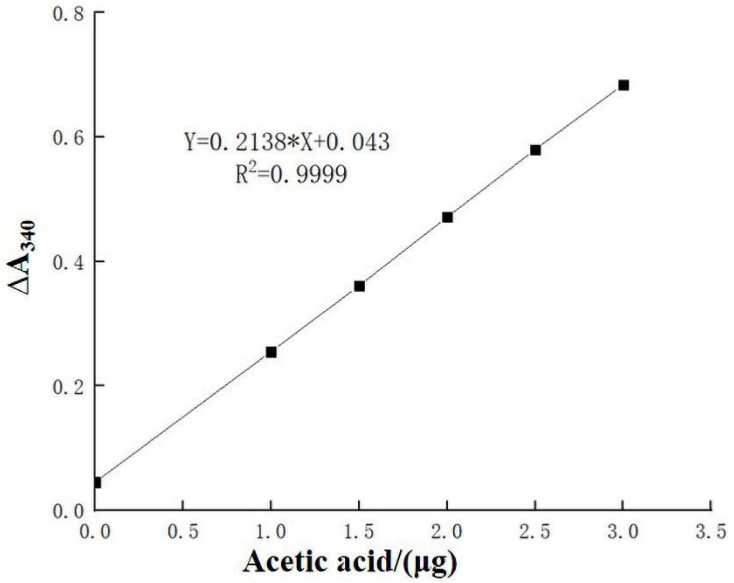

Figure S2 Standard curve of acetic acid for enzyme assay of *As*CDA.

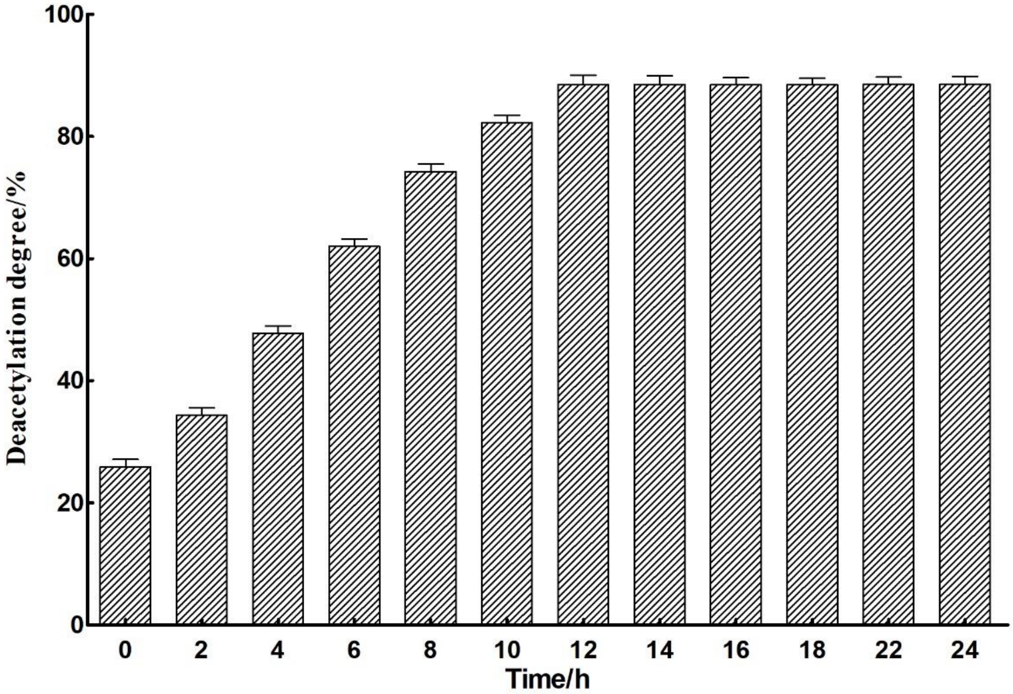

Figure S3 The determination of the degree of deacetylation of α-chitin treated by *As*CDA with different time points (0 h, 2 h, 4 h, 6 h, 8h, 10 h, 12 h, 14 h, 16 h, 18h, 22

h and 24 h.

Supplementary informations:

Amino acid sequences of 14 members of the deacetylase superfamily were shown below to perform the multiple sequence alignment.

>MW295944.1 [*Acinetobacter schindleri* MCDA01]

VTTAYLRGKQLIETIQKQEYSRDLIGYHGKPPHAQWPNAARIAVQFVLNY EEGGENHVEHGDSSSEQFLSEIVGAASFPAVHRSMDSMYEYGSRAGFWRIHEE FQKRGWPMTIFGVGMALARNPYIVEAIKAADYDVVSHGQRWLHYQDMEIET ERQHMDQALSVLTELFGETPIGWYTGRDSPNTRQLLAEFSQIKYDSDYYGDD LPFWSTLTEPNGQKRPHLIIPYTLECNDMKFSSPGGFNSGEQFYQYLKDAFDV LYSEGETAPKMMSIGMHCRLLGRPGRFKALQRFMDYVQSHDKVWICCRNDI AQHWYTHHFPAHQD

active sites: Glu^{51}, Glu^{52}, His^{140}, Trp^{144} and His^{276}; Glu^{51}: base catalytic site; His^{276}: acid catalytic site.

>APH81274.1 [*Pestalotiopsis* sp.]

MLAPLFAALLAGAATASPIQERQSSVPVGTIITACTVPNTFALTFDDGPFAY TSELLDLLSSNGVKATFFLNGQNWGSIYDYTSVVTRMDAEGHQIGSHTWSHA DLATLDAAGITSQMTQLETALTSILGKVPTYMRPPYFSTNALALSTLGGLGYH VINANIDTLDYEHDDDTIGVAFTNFQNGLASGGTVSLMHDVHAQTVHVLVQE AINAIKAKGLTPVTVGTCLGDASANWYKSGGGSGTTPPPATGGPSPDDTCGGS NGYVCQNSQCCSQWGWCGTTSEYCAAGCQAAYGPCT

>CAP60162.1 [*Podospora anserina*]

MINVPVTLLSLSAVATAAALADANSQSTSPNPSPRQHARDTLQKRAN
CGAGIGNCPSGQCCSQYGWCGVTSEHCGTGCQSGFGTCTGGGGGNNEE
TLSTPRPKFGSIPYGVTITNCNAAGTIALTFDDGPFLYTNQLLDLLAQQQV
KATFFTNGLNWGDATQAPYPDVLRRIVNDGHQLGSHTYNHPDLNTLTTA
ARRSNMAQNEKIFKDALGGYFPTYMRPPYGSCTGQCLTDLGDLGYHVIN
WNIDTLDYQGNIPNSQNIFNSAVSTNAAANKYIALAHDVHQATVQQLAL
GLIQTAKNRGYRLVTVGECLGDAPVNWYRDATTGNARTGGGGGSGGNP
NPGPITSTNGLCGSTNGNMNCLNSGFGNCCSQWGFCGSTAEYCGANCQR
AFGNCN

>EAA66447.1 [*Aspergillus nidulans* FGSC A4]

MFATLALVFTALASNALTTPLPLVRRVPTGQVITQCTTPNTIALTFDD
GPSEYTPQLLDLLSRYSARATFFVLGDAAAQNPGLLQRMRDEGHQVGA
HTYDHVSLPSLGYDGIASQMTRLEEVIRPALGVAPAYMRPPYLETNELVL
QVMRDLDYRVISASVDTKDYENQDADAIINTSFQLFLDQLDAGGNIVLA
HDIHYWTVASLAERMLQEVNARGLIATTVGDCLGDGEIAWYH

>QNP99526.1 [*Yarrowia lipolytica*]

MIWRALFLFVLPVATQANFTVKDLLQHKDNEWWAPCSAQGGTLDS
QQSIMAAEPFPPWLTAFTGLTAWPQDNPPYIPLDYVDLATVPNHIAKREL
GICDGVERTACSFDCHLCIAFDDIRTCNKISQTFDDGPSPSTPKLLEMLPSK
TTFFVQGVNVVRFPEIFREQHRQGHLLASHTWSHPNLASLSNEEIVAQLQ
WTNWAMNATAGIIPRYFRPPYGAIDNRVRAIVRMLGMQSVLWDRDTFD
WKVNAGIKTSPEVVEEVQDWKLQGGGWGLILEHDTTIKTVNVGLDVAK

ALGPNQLTVAECVGQTQWYQDPARLGNEPRRGHRAASYQRS

>BAP71097.1 [*Kluyveromyces marxianus*]

MILTCWIVNIWISIVLVSAAAIDMLDVESNSLSISAEDASKEATLSEQSIFPI
GRSPRSQAKTPFPAWLTQFTGLTEWPGANPPYIPLDFIDFNKIEKYEPYKEGRC
NKNPRESCSFDCHKCVAHDDVHSCAKLSQTFDDGPSVHTLTLLDKLQHKTTF
FTLGMNVVERPEIYHTVMERGHLIGTHTWSHPFLPSLTNEQIIAQLEWSIWAM
NATGNHLPKWWRPPYGGIDNRVRSIARQFGMQAVLWDHDTLDWLAASNPFQ
RTESAILKDVTEWKRTSNGLILEHDGSKKTVDIAMKVNDIIGSDQMTAAQCV
GGIDYIKEFKV

>NP_013411.1 [*Saccharomyces cerevisiae* S288C]

MRIQLNTIDLQCIIALSCLGQFVHAEANREDLKQIDFQFPVLERAATKTPF
PDWLSAFTGLKEWPGLDPPYIPLDFIDFSQIPDYKEYDQNHCDSVPRDSCSFD
CHHCTEHDDVYTCSKLSQTFDDGPSASTTKLLDRLKHNSTFFNLGVNIVQHP
DIYQRMQKEGHLIGSHTWSHVYLPNVSNEKIIAQIEWSIWAMNATGNHTPKW
FRPPYGGIDNRVRAITRQFGLQAVLWDHDTFDWSLLLNDSVITEQEILQNVIN
WNKSGTGLILEHDSTEKTVDLAIKINKLIGDDQSTVSHCVGGIDYIKEFLS

>KJD58109.1 [*Bacillus amyloliquefaciens*]

MKWFCSICCMAVLLAGGINSAEAVPNERINWGFKRSVNHQPPDAGRQLD
NLVKKYDAFYLGNTKEKTIYLTFDNGYENGYTPKVLDVLKKHHVTGTFFVTG
HFVKEQPDLIKRMSKEGHIIGNHSFHHPDLTTKTGDEIQDELDSVTEAVYNITG
KRDNLYLRPPRGVFSEYVLKETKRLGYQTVFWSVAFVDWKVNEQKGKQYAY
DHMIKQAHPGAIYLLHTVSKDNSEALDDAITDLKKEGYTFKSIDDLMFEKEM

KLPRL

>WP_019713628.1 [*Bacillus spizizenii*]

MKWMCSICCAAVLLAGGAAQAGAVANEPINWGFKRSVNHQPPDAG
KQLNRLIEKYDAFYLGHTKEKTIYLTFDNGYENGYTPKVLDVLKKHRVT
GTFFVTGHFVKDQPELIKRMSDEGHIIGNHSFHHPDLTTKTADQIQDELDS
VNEEVYKITGEQDNLYLRPPRGVFSEYVLKETKRLGYQTVFWSVAFVDW
KINNQKGKKYAYDHMIKQAHPGAIYLLHTVSRDNAEALDDAITDLKKQ
GYTFKSLDDLMFEKEMKLPSL

>AFQ56715.1 [*Bacillus subtilis* QB928]

MAMKWMCSICCAAVLLAGGAAQAEAVPNEPINWGFKRSVNHQPPD
AGKQLNSLIEKYDAFYLGNTKEKTIYLTFDNGYENGYTPKVLDVLKKHR
VTGTFFVTGHFVKDQPQLIKRMSDEGHIIGNHSFHHPDLTTKTADQIQDE
LDSVNEEVYKITGKQDNLYLRPPRGVFSEYVLKETKRLGYQTVFWSVAF
VDWKINNQKGKKYAYDHMIKQAHPGAIYLLHTVSRDNAEALDDAITDL
KKQGYTFKSIDDLMFEKEMRLPSL

>pdb:1W1A [*Bacillus subtilis*]

GPLGSVLLAGGAAQAEAVPNEPINWGFKRSVNHQPPDAGKQLNSLI
EKYDAFYLGNTKEKTIYLTFDNGYENGYTPKVLDVLKKHRVTGTFFVTG
HFVKDQPQLIKRMSDEGHIIGNHSFHHPDLTTKTADQIQDELDSVNEEVY
KITGKQDNLYLRPPRGVFSEYVLKETKRLGYQTVFWSVAFVDWKINNQK
GKKYAYDHMIKQAHPGAIYLLHTVSRDNAEALDDAITDLKKQGYTFKSI
DDLMFEKEMRLPSL

>NP_001342829.1 [*Schizosaccharomyces pombe*]

MYETRDLTGNAGKPVDTNPWPNNSKIAVSFVVNYEEGGERSLLYEDEGF
ETFLTEAGLMPFPNRPVRERSIESCFEYGSRCGFWRILNLFKKHKVPFTCWAIG
QAVEKNPVVVGAMEEAGCEVGSHSHRWINYEGVPPETEYEHIKKSVQAIQKA
SPSNSAPRSWYTGRASLNTRKLVCQVYKDLGLPQPFDSDEYNDDYPYWVAD
PLASKPGAEDDKGLLIVPYTLEVNDMKYAVAPGFCNSDDFYTYARDAFDVLY
EEGLEGAPKMMTIGLHCRLTGRPGRFRGLQKLMEHITSKEGVWVATREQIAQ
AWSAKHPYKA

>pdb:3CL6 [*Pseudomonas fluorescens*]

MSVDYPRDLIGYGSNPPHPHWPGKARIALSFVLNYEEGGERNILHGDKE
SEAFLSEMVSAQPLQGERNMSMESLYEYGSRAGVWRILKLFKAFDIPLTIFAVA
MAAQRHPDVIRAMVAAGHEICSHGYRWIDYQYMDEAQEREHMLEAIRILTEL
TGERPLGWYTGRTGPNTRRLVMEEGGFLYDCDTYDDDLPYWEPNNPTGKPH
LVIPYTLDTNDMRFTQVQGFNKGDDFFEYLKDAFDVLYAEGAEAPKMLSIGL
HCRLIGRPARLAALQRFIEYAKSHEQVWFTRRVDIARHWHATHPYTGAAK

>ARW18113.1 [*Komagataeibacter europaeus*]

MSGTGVYPRDLVGYGGNPPDAKWPGGARIAVQFVINYEEGAENSVLHG
DRGSEAFLSEMIGAQSITGARAIAMESLYEYGSRAGFWRLHRIFTQRKQPLTVF
GVAAAMARNPAAVAAMKEAGWEIASHGLRWIDYQHVPEAVERAHIQECIAL
HTKLTGSRPLGWYQGRTSPNTARLIAEEGGFVYDADSYADDLPYYDRSHGRA
QLIVPYTLDVNDMRFVAINGFTEGEQFFTYLRDTFDELYEEGADRPRMMSVG
LHCRVAGRPGRARAVARFLDYIATREKVWVVTRLDIARHWLETHPA

>CDI21894.1 [*Klebsiella pneumoniae*]

MGENQEHYPRDLRGYAGEPPHARWPGGARIAVQFVLNYEEGAENH

VLHGDAGSEQFLSDIIGAASYPARHMSMDSLYEYGSRAGFWRIHREFSQR

GLPLTVFGVAMALARHPEIVAAIKAADYDVVSHGWRWIHYQHMDIAEER

EHLQKAVQVLTDLFGKPPTGWYTGRDSPNTRQLVVEHGGFDYDSDYYG

DDLPFWSEVACSDGSQRPHLIVPYTLDANDMRFATAQGFNTAEQFYTYL

KDSFDVLYAEGETAPKMMSVGMHCRLLGRPGRFRALQRFLDYIQQHDK

VWVCTRQQIADHWRETHPYRG
